# The impact of the COVID pandemic on the attitude and mental health of healthcare professionals working in COVID departments

**DOI:** 10.1192/j.eurpsy.2022.1385

**Published:** 2022-09-01

**Authors:** O. Elleuch, R. Feki, I. Gassara, N. Smaoui, N. Charfi, S. Omri, M. Maalej, J. Ben Thabet, L. Zouari, M. Maalej

**Affiliations:** 1 Hedi Chaker Hospital, Sfax, Psychiatry C, Sfax, Tunisia; 2 Hedi Chaker University Hospital, Psychiatry, Sfax, Tunisia; 3 Hedi Chaker University Hospital, Psychiatry C, Sfax, Tunisia

**Keywords:** Anxiety, Healthcare professionals, Covid-19, Depression

## Abstract

**Introduction:**

The COVID pandemic brought an overwhelming impact on hospital systems and personnel in the world.

**Objectives:**

Our study aimed to examine the impact of the COVID pandemic on the attitude and mental health of healthcare professionals working in COVID departments.

**Methods:**

We included 23 healthcare professionals working in the COVID departments of the Hospitals of Sfax. Sociodemographic data, medical history and COVID related details were collected from the participants. Their mental health was assessed by the Hospital anxiety and depression scale (HADS).

**Results:**

In our sample, 26.1% were men and 73.9% were women, they were aged from 26 to 52. They worked for 57 hours per week, including 27.38 hours of direct contact with COVID positive patients and 5.43 nightshifts per month. A rate of 52.2% of the participants claimed having enough protection tools and 13% confirmed having enough equipment for patient management. 30.4% feared the lack of equipment and 39.1% considered themselves trained enough to manage COVID patients. As for the protective measures, 95.7% reduced contact with family members, 30.4% stopped visiting their parents, 8.7% left the family house and 4.3% didn’t take any particular measure. The mean HADS depression score was 9.61, and 60.86% had a Depression score equal or greater than 8, indicating depression. As for the HADS Anxiety score, its mean was 10.61 and 69.56% had a score equal or greater than 8, indicating anxiety.

**Conclusions:**

The pandemic had a big impact on healthcare professionals working in COVID departments, as shows the relatively high depression and anxiety rate.

**Disclosure:**

No significant relationships.

